# Mixed‐complexity artificial grammar learning in humans and macaque monkeys: evaluating learning strategies

**DOI:** 10.1111/ejn.12834

**Published:** 2015-03-01

**Authors:** Benjamin Wilson, Kenny Smith, Christopher I. Petkov

**Affiliations:** ^1^ Institute of NeuroscienceNewcastle UniversityHenry Wellcome BuildingFramlington PlaceNewcastle upon TyneNE2 4HHUK; ^2^ Centre for Behaviour and EvolutionNewcastle UniversityNewcastle upon TyneUK; ^3^ School of Philosophy, Psychology and Language SciencesUniversity of EdinburghEdinburghUK

**Keywords:** artificial grammar learning, Rhesus macaques, rule learning, statistical learning

## Abstract

Artificial grammars (AG) can be used to generate rule‐based sequences of stimuli. Some of these can be used to investigate sequence‐processing computations in non‐human animals that might be related to, but not unique to, human language. Previous AG learning studies in non‐human animals have used different AGs to separately test for specific sequence‐processing abilities. However, given that natural language and certain animal communication systems (in particular, song) have multiple levels of complexity, mixed‐complexity AGs are needed to simultaneously evaluate sensitivity to the different features of the AG. Here, we tested humans and Rhesus macaques using a mixed‐complexity auditory AG, containing both adjacent (local) and non‐adjacent (longer‐distance) relationships. Following exposure to exemplary sequences generated by the AG, humans and macaques were individually tested with sequences that were either consistent with the AG or violated specific adjacent or non‐adjacent relationships. We observed a considerable level of cross‐species correspondence in the sensitivity of both humans and macaques to the adjacent AG relationships and to the statistical properties of the sequences. We found no significant sensitivity to the non‐adjacent AG relationships in the macaques. A subset of humans was sensitive to this non‐adjacent relationship, revealing interesting between‐ and within‐species differences in AG learning strategies. The results suggest that humans and macaques are largely comparably sensitive to the adjacent AG relationships and their statistical properties. However, in the presence of multiple cues to grammaticality, the non‐adjacent relationships are less salient to the macaques and many of the humans.

## Introduction

Understanding which brain processes are evolutionarily conserved in humans and other animals, and which have undergone unique specialisation in humans requires cross‐species comparisons. However, neurobiological studies depend on behavioural insights into auditory or language‐related processing in humans and other species.

Artificial grammar (AG) learning paradigms have shown that human and non‐human animals can process certain relationships between elements in a sequence (Fitch & Hauser, 2004; Gentner *et al*., 2006; Saffran *et al*., 2008; Wilson *et al*., 2013). The complexity of these relationships can be controlled experimentally, related to features of human language or animal song, and quantitatively compared with other sequence‐processing paradigms (Petkov & Wilson, 2012; Wilson *et al*., 2013), such as auditory oddball or rhythm perception paradigms (Ulanovsky *et al*., 2003; Selezneva *et al*., 2013). Moreover, human neuroimaging studies have shown that AG learning tasks can engage certain regions in the perisylvian (fronto‐temporal) language network (Petersson *et al*., 2004; Friederici *et al*., 2006). In this paper, we directly compare the sensitivity of macaques and humans with various features of a mixed‐complexity AG to inform neurobiological research.

Artificial grammars generate rule‐based sequences of stimuli, regulating how the stimulus elements in a sequence are ordered and establishing relationships between the constituent elements (Reber, 1967). In AG learning paradigms, participants are typically exposed to exemplary sequences generated by the AG, then tested with sequences that are either ‘consistent’ with the AG or that ‘violate’ it. Different behavioural responses to violation vs. consistent sequences can provide evidence that the participant learned something about the sequences generated by the AG. This approach has been used to obtain evidence for AG learning in adult humans, pre‐linguistic infants and a number of non‐human species (Reber, 1967; Marcus *et al*., 1999; Fitch & Hauser, 2004; Friederici, 2004; Gentner *et al*., 2006; Wilson *et al*., 2013).

While different responses to consistent and violation sequence are suggestive of AG learning, they may be insufficient to identify the specific processes or learning strategies employed, which could differ both between and within species (van Heijningen *et al*., 2009). Moreover, while many studies have tested the learning of different AG rules in separate experiments (Fitch & Hauser, 2004), natural language and certain animal songs often have multiple levels of complexity, containing both adjacent (local) and non‐adjacent (longer‐distance) relationships. Mixed‐complexity AGs allow us to simultaneously evaluate sensitivities to different features of an AG, and therefore to assess whether some features or properties of the AG may be more salient than others (Romberg & Saffran, 2013).

We used a mixed‐complexity AG (based on Saffran *et al*., 2008) that we have previously studied in non‐human primates (Wilson *et al*., 2013). Here, we test adult humans and Rhesus macaques using a broad range of AG sequences containing both adjacent and non‐adjacent relationships to assess the learning strategies of both species. Insights on behavioural capabilities and sequence‐processing strategies that different species adopt are important for interpreting neurobiological findings and addressing which aspects of human behaviours can be modelled in non‐human primates.

## Materials and methods

### Ethics statement

All animal work and procedures performed were approved by the Animal Welfare and Ethical Review Body at Newcastle University, and by the UK Home Office. The work complies with the Animal Scientific Procedures Act (1986) on the care and use of animals in research, and with the European Directive on the protection of animals used in research (2010/60/EU). We support the principles on reporting animal research stated in the consortium on Animal Research Reporting of *In Vivo* Experiments (ARRIVE). All persons involved in this project were Home Office certified and the work was strictly regulated by the UK Home Office. Human participants provided informed consent to participate in this study, which was approved by the human studies Ethical Review Body at Newcastle University and which conformed with the 2013 WMA Declaration of Helsinki.

### Stimuli

**Figure 1:**
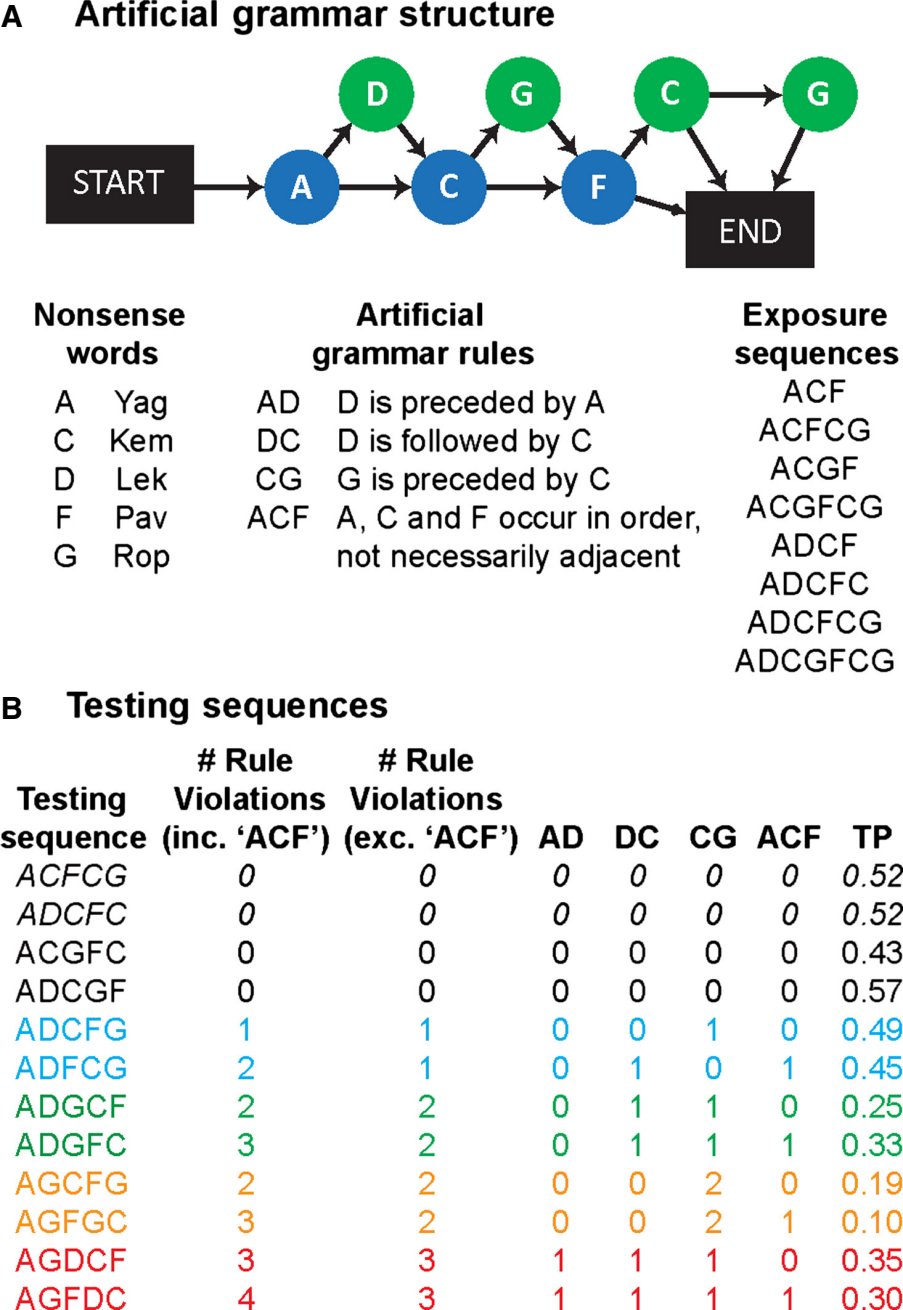
Artificial grammar (AG) and stimulus sequences. (A) The AG contains five unique elements. Sequences (strings of nonsense words) consistent with the AG are generated by following any path of arrows from START to END. Violation sequences do not follow the arrows. The AG generates consistent sequences; however, all legal sequences must follow any of a number of ‘rules’, see text. The AG was used to generate eight exposure sequences, which follow all of these rules. Each experiment began with an exposure phase where the human or monkey participants passively listened to the habitation sequences for 5 or 30 min, respectively. (B) Four ‘consistent’ testing sequences (black) were generated from the AG. The first two of these sequences (in italics) were also presented in the exposure period (familiar), while the second two were novel to the testing phase of the experiments. Each consistent sequence was presented twice in each testing run, to balance the number of consistent and violation sequences presented. Eight violation sequences were generated, including different rule violations and a range of transitional probabilities (TPs; see Materials and methods). The sequences were designed in four pairs (denoted by colours). These pairs were matched as closely as possible for the rule violations they included and for their average TPs. Moreover, the second sequence in each pair, but not the first, violated the non‐adjacent, long‐distance, ‘ACF’ rule.

The stimuli in the Rhesus macaque and the human experiments were identical. Each of the stimulus sequences (Fig. 1B) was created by digitally combining recordings of naturally spoken nonsense words produced by a female speaker based on an AG (Fig. 1A) developed by Saffran (2002) and (Saffran *et al*., 2008). Nonsense words were selected as stimuli because they have the advantage of being spectro‐temporally complex stimuli that are easy to distinguish from each other. The nonsense words were recorded with an Edirol R‐09HR (Roland) sound recorder. The amplitude of the recorded sounds was root‐mean‐square balanced. We computed the power spectra of the nonsense word stimuli and confirmed that they fall well within the auditory ranges of both species (Fig. S3). The nonsense word stimuli were randomly assigned to the AG elements (i.e. A = ‘yag’, C = ‘kem’, etc.). They were then combined into exposure and testing sequences using customised Matlab scripts (150 ms inter‐stimulus intervals). All the nonsense words were duration matched (413 ms), and each test sequence contained five nonsense word elements (sequence duration = 2665 ms).

The AG can generate 12 legal sequences that are consistent with the AG. To ensure the participants could be tested with novel consistent sequences, which they had not previously been exposed to, eight consistent sequences were selected for the exposure phase of the experiment (Fig. 1B). The participants were then tested with four consistent sequences including two familiar and two novel sequences (Fig. 1B). To ensure that the number of presentations of consistent and violation sequences (see below) were balanced, each of the consistent test sequences was presented twice in each testing run. We confirmed that the reported results (below) were also evident when we only analysed responses to the first presentation of each consistent sequence (see Supporting Information).

The AG used in this experiment contains a number of ‘rules’ that all legal, consistent sequences must follow (Fig. 1A). Adjacent rules are such that if ‘D’ is present it must be preceded by ‘A’; ‘D’ must be followed by ‘C’; each ‘G’ must be preceded by ‘C’. The AG also contains three obligatory elements (‘A’, ‘C’ and ‘F’), which must occur in every sequence in the order ‘A’, ‘C’, ‘F’, but not necessarily next to each other (non‐adjacent ‘ACF’ rule). We generated eight ‘violation’ sequences that each violated at least one of these rules. The sequences contained a range of different rule violations, in order to investigate whether the participants might respond more strongly to sequences containing higher numbers of violations (Fig. 1B).

A key aim of this study was to test participants’ sensitivity to the non‐adjacent ‘ACF’ rule (Fig. 1A). In this AG it is not possible to violate this non‐adjacent rule without also violating at least one of the adjacent rules; for example, the ‘C’ element cannot occur before the ‘A’ element without creating an illegal adjacent transition. In order to determine if sensitivity to the ‘ACF’ rule violation goes beyond sensitivity to the adjacent violations, the violation sequences were designed in pairs. The pairs were balanced for the number of adjacent rule violations that they include, wherever possible on the specific rules violated, and on their average transitional probabilities (TPs). Importantly, one of the pairs of comparison sequences also included a violation of a non‐adjacent relationship between the key ‘A’, ‘C’ and ‘F’ elements (Fig. 1B). Different responses to the sequences containing the non‐adjacent violations, relative to the comparison sequences that did not have this violation, would suggest that a participant was sensitive to the non‐adjacent relationship.

In addition to sensitivity to the rules of the AG, we considered the statistical properties of the sequences. The statistical probability of each transition between elements (TP) was calculated as follows:

$$\mathrm{TP}\;\mathrm{of}\;X\;\mathrm{to}\;Y\;=\;P\left(Y|X\right)\;=\;\frac{\mathrm{frequency}\;\mathrm{of}\;XY}{\mathrm{frequency}\;\mathrm{of}\;X}$$

A TP of 1 denotes a transition that must always occur, a ‘rule’ (e.g. ‘D’ must always be followed by ‘C’; Fig. 1A), and a TP of 0 represents an illegal, violation transition, not permitted by the AG. Some transitions between elements, while legal, occur more or less frequently than others. Therefore, the average TPs of a sequence reflect the statistical likelihood of the elements in a sequence occurring in that order. If participants are sensitive to these statistical properties established during the exposure phase, behavioural responses should correlate with the TPs of the sequences. A ‘rule’ represents a relationship that must occur in a legal sequence (a transition with a TP of 1), and violating this rule produces an illegal transition (with a TP of 0). However, it is possible to create illegal transitions that do not violate any of these rules. For example, the ‘A’ element can be legally followed by either ‘C’ or ‘D’, therefore there is no fixed ‘rule’ about what can follow ‘A’. However, the transition from ‘A’ to ‘F’ is illegal, and would have a TP of 0. Therefore, although rule violations and TPs are inherently related, they are not perfectly correlated (Spearman's *r *= −0.386; *P *=* *0.345): beyond simply representing the number of rule violations, the average TP of a sequence considers the combined probability of every pair‐wise transition between elements. It is thus important to note that TPs are sensitive to the adjacent relationships (transitions between elements) but are not sensitive to non‐adjacent transitions (i.e. the average TP of sequences that violate the non‐adjacent ‘ACF’ rule is 0.30, which is very similar to those sequences consistent with the rule, 0.32; Fig. 1B).

### Rhesus macaque experiment

#### Participants

Two adult male Rhesus macaques (*Macaca mulatta*) from a group‐housed colony participated in this experiment (ages: M1 = 14 years, M2 = 6 years; weights: M1 = 10 kg, M2 = 16 kg). Prior to testing, the animals had controlled access to fluid, so that the juice that they obtained for correctly completing the task was sufficiently rewarding. Every individual is different, thus our fluid control procedure was individually customised (for a review, see: Prescott *et al*., 2010), to a level that just motivates that particular individual to complete their task while maintaining their normal health and physiology. Both animals were previously trained on a fixation task and slowly acclimated to head immobilisation with positive reinforcement, to allow eye‐tracking data to be obtained.

Each macaque participated in 16 eye‐tracking testing runs (see ‘Procedures’, below). Our sample size is constrained by the ethical need to study the fewest non‐human animals possible to obtain statistically robust results, and the need to study the animals with a method sensitive enough to measure effects in individual animals (in this case 16 eye‐tracking testing runs in a laboratory setting). In a previous study we have tested a larger group of macaques using more traditional approaches, coding video‐recordings of natural orienting responses, but have found this approach to have insufficient power to evaluate learning in individual macaques (Wilson *et al*., 2013). Eye‐tracking offers a more objective assessment of eye‐orienting responses, which, although sensitive enough to address what each animal is looking at, also requires considerable amounts of data given the variability in natural looking responses (Wilson *et al*., 2013).

#### Procedures

**Figure 2:**
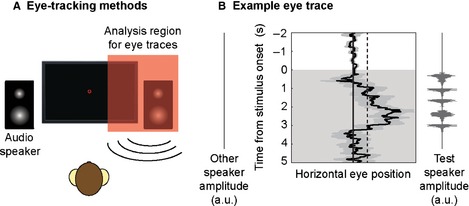
Details of macaque eye‐tracking approach. (A) Schematic of macaque eye‐tracking experiment, adapted from Wilson *et al*. (2013). (B) Average eye trace (± SEM) from an example session in one monkey. Values to the right of the vertical midline represent eye movements toward the audio speaker (left or right) that presented the test sequence. The 2 SD baseline (based on the variance in eye movements during the 2 s baseline period) is shown as a dashed line. The duration for which the eye position exceeded this threshold during the 5 s stimulus period (shaded area) was calculated.

The experiment was performed in a customised sound‐attenuating chamber (IAC Acoustics). Animals were tested individually. The macaque was seated in a primate chair 60 cm in front of a computer monitor (which displayed a yellow fixation circle) and two audio speakers (Creative Inspire T10) horizontally positioned at ± 30° visual angle (Fig. 2A). During the testing phase of the experiment, stimulus sequences were presented from either the left or the right audio speaker while eye‐tracking data were recorded (220 Hz infra‐red eye‐tracker; Arrington Research; Fig. 2B). The sounds were presented using Cortex software (Salk Institute) at ~75 dB SPL (calibrated with an XL2 sound level meter; NTI Audio). For additional details of the eye‐tracking procedure, see Wilson *et al*. (2013).

In each animal, testing took place over several testing sessions on separate days. Each testing session consisted of several testing runs that were each preceded by either an exposure or a refamiliarisation phase. Therefore, each testing session took the form: exposure phase, testing run, refamiliarisation phase, testing run, refamiliarisation phase, testing run, etc.

#### Exposure and refamiliarisation phases

Each testing session began with an exposure phase, during which the exposure sequences were presented in a random order over both audio speakers for 30 min while the monkey passively listened to the sequences (Fig. 1B; 288 sequences; 36 presentations of each sequence; rate of ~10 sequences/min; inter‐sequence interval = 3 s). The exposure phase was then followed by a testing run (see next section). Subsequent testing runs were separated by a refamiliarisation phase, which was identical to the exposure phase, except that its duration was 5 min. Eye‐tracking data were not recorded during the exposure and refamiliarisation phases, as useful eye‐tracking data depended on the monkeys starting each trial by fixating to centre the eyes and to establish a baseline looking response to use for analysis. We reasoned that if we had required the animals to fixate for a juice reward during the ~300 exposure trials, they would have been satiated before the start of testing and may not have completed sufficient numbers of testing runs.

#### Testing runs

Each testing run consisted of multiple fixation trials. The task was self‐paced such that a fixation spot was presented in the centre of the screen and the testing trial only began when the monkey fixated upon the spot. If the monkey continuously fixated for 2 s the trial continued, otherwise the trial was aborted and a new trial began after a 3 s inter‐trial interval. To maintain the novelty of the stimulus presentations, and to encourage the macaques to look towards the speakers, only 25% of successful fixation trials were followed by the presentation of a test sequence (Fig. 1B) from either the right or the left audio speaker (Fig. 2A). The trials on which a testing sequence was presented were separated by, on average, four trials where no test sequence was presented and the animal received a juice reward immediately after fixating. The trials on which stimuli were presented, eye‐tracking data were recorded for a further 5 s (testing sequence duration = 2665 ms, total eye data recording period = 7000 ms; Fig. 2B). A juice reward was delivered after this period, to reward the monkey for correctly completing the fixation trial, regardless of the type of testing sequence that was presented; no feedback or reward was given for responding to any of the sequences.

Testing sessions, which were conducted on different days, consisted of two to four separate testing runs (macaque 1 participated in four testing sessions, each consisting of four testing runs; macaque 2 participated in five testing sessions: two sessions contained four testing runs, two sessions contained three testing runs and one session contained two testing runs). Each testing run lasted ~15 min, and testing sessions lasted ~2 h.

#### Data analysis

The eye‐tracking data for each trial contained both a 2 s baseline period during which the animal fixated on the central fixation spot, and a subsequent 5 s stimulus period during which the test sequence was presented (Fig. 2B). To calculate the duration of looking responses towards the presenting audio speaker, we initially calculated the baseline variability in the eye movement during the 2 s fixation period of each trial. Looking responses to the test sequences were defined individually for each animal as looks toward the presenting audio speaker (left or right) exceeding 2 SD of the variability in the baseline fixation period. When the data were analysed using a different threshold (e.g. 3 SD) or timing window (e.g. 4 s following the stimulus presentation rather than 5 s; see Fig 2B), comparable results were obtained. For additional methodological details, see Wilson *et al*. (2013).

### Human experiment

#### Participants

Thirty‐three human participants (age range 18–30 years, median age of 20 years; 23 female, 10 male) were recruited through the Newcastle University Institute of Neuroscience participation scheme and provided informed consent to participate. It is not feasible to test human participants for as many sessions as our macaques; therefore, it was necessary to test a larger group of participants. The number of data points (i.e. testing sessions) from the 33 human participants is approximately equal to those obtained from the two macaques (each completing 16 testing runs; 32 testing runs in total). All human participants were native English speakers, and reported normal hearing and normal or corrected‐to‐normal vision. No participants reported any language or comprehension disorders in a pre‐study questionnaire.

#### Procedures

The human participants were tested individually in a psychophysics testing laboratory. Participants were seated 1 m in front of a computer monitor. Stimuli were presented through Denon AH‐D 310R headphones at ~75 dB SPL. Responses were made by pressing one of two keys on the keyboard. The experiment was run using custom Matlab scripts (Psychophysics Toolbox: http://psychtoolbox.org). In total each participant took part in five testing runs, each preceded by either an exposure or refamiliarisation phase.

#### Exposure phase

During the initial exposure phase, the participants were asked to listen to the exposure sequences (Fig. 1A) for 5 min (48 sequences; six presentations of each exposure sequence; rate of ~10 sequences/min; inter‐sequence interval = 3 s). Subsequent refamiliarisation phases presented the same exposure sequences, in a randomised order, for 3 min (32 sequences; 4 presentations of each exposure sequence).

#### Testing phase

Following each exposure phase was a testing phase during which the testing sequences (eight violation sequences and two presentations each of the four consistent sequences; Fig. 1B) were presented twice in a random order, for a total of 32 trials. Following the presentation of each sequence, a circle on the computer monitor changed from blue to yellow, indicating that the participant should respond either that the sequence ‘followed the same pattern’ as the exposure sequences (consistent) or that it ‘did not follow the pattern’ (violation). The participants were not allowed to respond during the presentation of a testing sequence, to ensure that their response was based on the whole sequence and not only on the first few elements. Therefore, reaction times reflected how quickly the participants responded following the end of the sequence presentation, and thus we did not find reaction times to the different stimulus conditions to be informative (see Supporting Information). Following the participant's response, the next trial began after an inter‐trial interval of 2 s.

#### Data analysis

To allow a closer comparison to the non‐human primate results, data are plotted as the proportion of trials to which the participants indicated that the sequences ‘did not follow the pattern’ (‘violation’ response; Fig. 4). Therefore for consistent sequences (blue in Fig. 4), responses below the 50% chance level indicate good performance, and for violation sequences (red in Fig. 4) responses above the chance level indicate good performance. This facilitates more direct comparisons between responses to consistent and violation test conditions across the species.

## Results

Before considering the results, we overview the key features of the AG and the experimental design, which are important for understanding the results obtained (see Materials and methods for details). The AG used here (based on Saffran *et al*., 2008) contains both obligatory and optional elements, which give it a non‐deterministic (less predictable) branching structure with considerable variability in the TPs between the elements (Fig. 1). This is also a mixed complexity AG because it contains both adjacent and non‐adjacent relationships between the elements. The obligatory elements, ‘A’, ‘C’ and ‘F’, must occur in every sequence and in that order, but not necessarily next to each other (as the optional elements can intervene; Fig. 1A). However, in this AG violations of non‐adjacent relationships also create illegal adjacent transitions. Thus, to address whether the participants were sensitive to non‐adjacent violations, it was necessary to balance the adjacent violations also created by violating the non‐adjacent ‘ACF’ relationship between sequences. Therefore, the eight violation sequences were designed in four comparison pairs. The pairs of sequences were matched in their adjacent violations; however, one of the sequences additionally contains the non‐adjacent ‘ACF’ violation (Fig. 1B). Differences between these comparison pairs of sequences provide evidence that the participants responded more strongly to the non‐adjacent violation, beyond the (matched) adjacent violations in the comparison sequence.

### Macaque experiment

The non‐human primates were initially exposed to the exemplary sequences generated by the AG (Fig. 1B). We then used infra‐red eye‐tracking to measure the durations of the two Rhesus macaques’ looking responses towards an audio speaker from which we presented the ‘consistent’ and ‘violation’ testing sequences in random order (see Materials and methods; Fig. 2).

**Figure 3:**
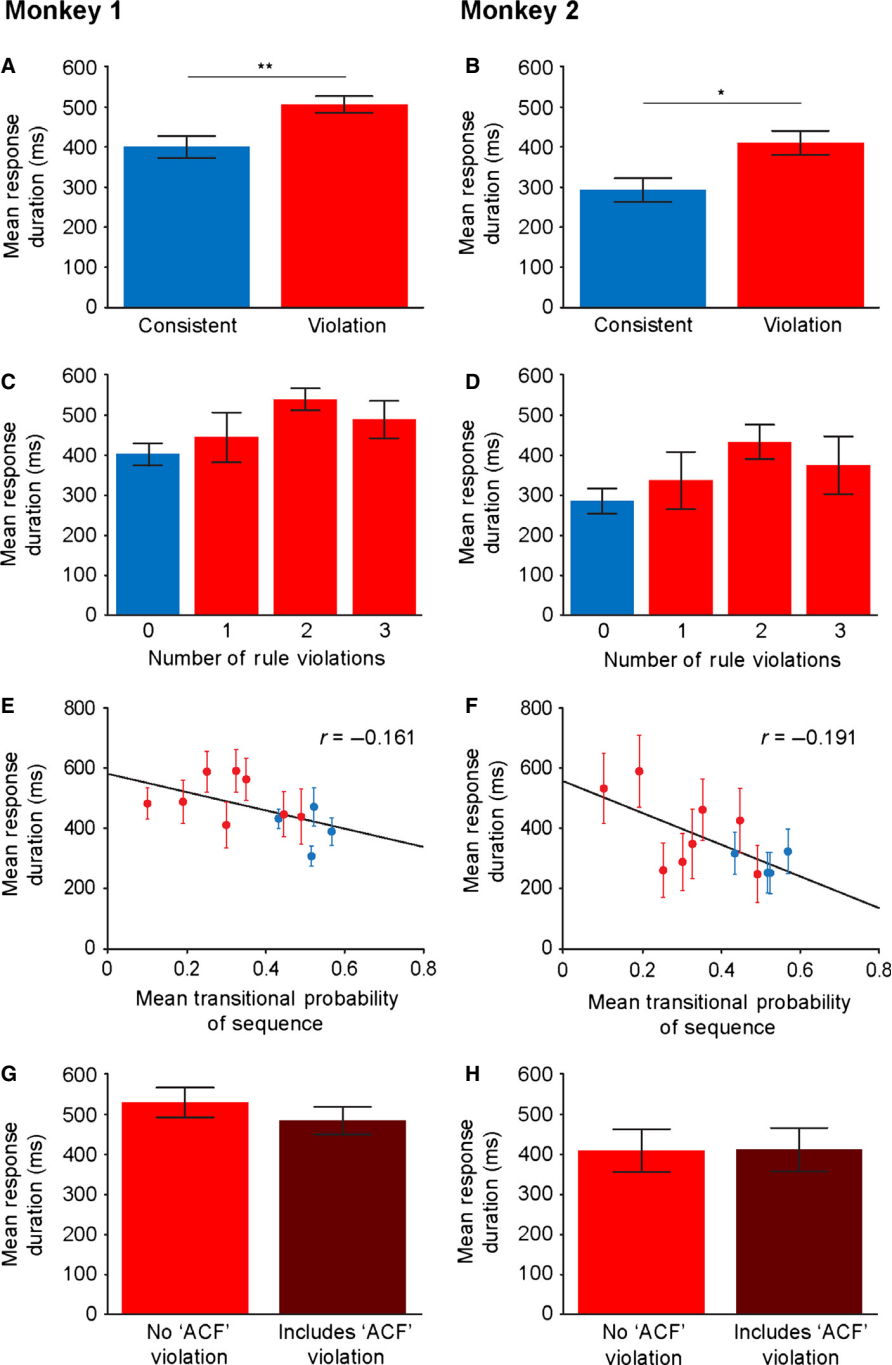
Monkey experiment results. (A and B) Mean (and standard error of the mean, SEM) looking response duration towards the presenting audio speaker to consistent and violation testing sequences in both macaques. (C and D) Mean (± SEM) response duration, separated based on the number of rule violations in the consistent (blue) or violation (red) sequences. (E and F) Mean (± SEM) response duration plotted against the mean transitional probability (TP) of each consistent (blue) and violation (red) sequence. (G and H) Mean (± SEM) response duration to violation sequences that only contained local violations but not the long‐distance, non‐adjacent ‘ACF’ relationship (red). This is contrasted to sequences that violate the long‐distance ‘ACF’ association in addition to matched local violations (dark red). *P <* *0.05, **P <* *0.01.

To investigate whether the macaques were sensitive to violations of the AG, we conducted a repeated‐measures (RM)‐anova with the dependent variable ‘response duration’, including the factors ‘condition’ (consistent and violation sequences) and ‘monkey’ (two levels). A main effect of ‘condition’ demonstrated that the monkeys responded more strongly to violations of the AG (*F*_1,30_ = 19.4, *P *<* *0.001). There was no interaction between ‘condition’ and ‘monkey’ (*F*_1,30_ = 0.54, *P* = 0.819), suggesting that the results are consistent between both animals. Similar results were observed when the responses of each animal were analysed individually (paired‐samples *t*‐tests, M1: *t*_15_ = 3.628, *P* = 0.002; M2: *t*_15_ = 2.839, *P *=* *0.012; Fig. 3A and B). To determine whether this effect could be attributed to the monkeys simply responding more strongly to sequences that were not present in the exposure phase, we conducted a second analysis separately comparing novel and familiar consistent sequences with the violation sequences (Fig. S2). An RM‐anova with the factors ‘condition’ (novel and familiar consistent sequences and violation sequences) and ‘monkey’ revealed a main effect of condition (*F*_2,29_ = 9.941, *P *=* *0.001) and no interaction between the factors (*F*_2,29_ = 0.213, *P *=* *0.809). Bonferroni‐corrected *post hoc* tests reveal no differences between the familiar and novel consistent sequences (*P *=* *1.0), but showed large differences between responses to the violation sequences and the familiar (*P *=* *0.001) and novel sequences (*P *=* *0.005), respectively. This analysis suggests that the observed sensitivity to violation sequences generalises to novel consistent sequences, which were not heard by the animals during the exposure phase. This is consistent with the generalisation that we have previously seen in macaques with a different version of this AG learning paradigm (Wilson *et al*., 2013). The results demonstrate that the macaques are sensitive to violations of the AG, and that their responses cannot be attributed solely to the familiarity of the test sequences. Also, additional analyses confirmed that the response to the novel stimuli was stable throughout the testing runs (Supporting Information).

To gain further insights into the pattern of macaque behavioural results, we next tested whether the animals responded more strongly to sequences containing higher numbers of rule violations. An RM‐anova with factors: ‘number of rule violations’ (four levels: 0, 1, 2 or 3 rule violations; Fig. 1B) and ‘monkey’ (two levels) showed a subtle but statistically significant main effect of ‘number of rule violations’, suggesting that the monkeys responded more strongly to sequences with higher numbers of violations (*F*_3,120_ = 2.847, *P *=* *0.04; Fig. 3C and D). There was no interaction between ‘number of rule violations’ and ‘monkey’, suggesting that this pattern is consistent between the animals (*F*_1,120_ = 0.004, *P *=* *1.0). These observations suggest that the macaques’ behavioural sensitivity scales with the number of rule violations present in the testing sequences, although interestingly the data also show a plateau of looking responses after two rule violations (Fig. 3C and D).

We next investigated whether the monkeys were sensitive to the statistical properties of the testing sequences. To do this we calculated the mean TP of the testing sequences (Fig. 1B), and tested the relationship between mean TP and behavioural responses. We conducted an ancova with the dependent variable: ‘response duration’, including the ‘TP’ of the sequences as a covariate and ‘monkey’ (two levels) as a between‐subjects factor. A strong main effect of ‘TP’ demonstrated that the durations of the monkeys’ responses were strongly negatively correlated with the statistical properties of the sequences, with longer responses to sequences with more uncommon transitions (*F*_1,380_ = 12.139, *P *=* *0.001; Fig. 3E and F). There was no interaction between the ‘monkey’ factor and ‘TP’ (*F*_1,380_ = 0.876, *P *=* *0.35), suggesting that both monkeys responded comparably. These results were supported by a partial regression between ‘mean TP’ and ‘response durations’, controlling for ‘monkey’, which showed a negative correlation (*r *= −0.176, *P *=* *0.001; Fig. 3E and F). Finally, separate correlation analyses in the individual animals found the same pattern of results (M1: *r *= −0.161, *P *=* *0.025; M2: *r *= −0.191, *P *=* *0.008). These results suggest that the monkeys’ responses are inversely dependent on the statistical properties of the AG sequences, with longer responses to sequences containing transitions that were uncommon in the exemplary AG sequences heard during the exposure phase.

These results suggest that macaques are sensitive to adjacent relationships in the AG. We next tested whether the monkeys were sensitive to violations of the non‐adjacent ‘ACF’ relationship, in addition to the adjacent violations. The violation test sequences were designed in four pairs, in which both comparison sequences contained the same number of adjacent violations and comparable mean TPs, but only one of the comparison sequences contained an additional violation of the non‐adjacent ‘ACF’ relationship (Fig. 1B). Longer responses to the sequences containing this additional ‘ACF’ violation would demonstrate sensitivity to this non‐adjacent relationship, over and above the matched adjacent violations. An RM‐anova with the factors: ‘sequence type’ (‘ACF violation’ or ‘no ‘ACF’ violation’), ‘sequence pair’ (four levels) and ‘monkey’ (two levels) was performed with the dependent variable of ‘response duration’. There was no main effect of ‘sequence type’ (‘ACF violation’ vs. ‘no ‘ACF’ violation’; *F*_1,30_ = 0.048, *P *=* *0.828, Fig. 3G and H), providing no evidence that the monkeys responded to the non‐adjacent ‘ACF’ violations, and suggesting that they were primarily sensitive to local, adjacent cues in this mixed‐complexity AG. There was a statistically significant main effect of ‘sequence pair’ (*F*_3,28_ = 3.342, *P *=* *0.033), showing that the monkeys responded more strongly to the pairs of sequences containing more rule violations and lower TPs, consistent with the results seen in Fig. 3C and D. Finally, there was no interaction between ‘sequence type’ and ‘sequence pair’ (*F*_3,28_ = 1.766, *P *=* *0.177), ‘monkey’ and ‘sequence type’ (*F*_1,30_ = 0.127, *P *=* *0.724), or ‘monkey’ and ‘sequence pair’ (*F*_3,28_ = 0.294, *P *=* *0.830).

This overall pattern of results demonstrates that the monkeys responded for significantly longer to violation sequences than consistent ones, and that this effect was more pronounced in sequences with lower TPs and higher numbers of adjacent rule violations. However, these results do not provide evidence that the macaques are sensitive to the non‐adjacent relationship also present in the sequences in this mixed‐complexity AG.

### Human experiment

Thirty‐three human participants were individually exposed to the same exposure sequences as the monkeys (Fig. 1B). Two experiments using eye‐tracking to measure the natural looking responses of the human participants failed to show any effects, even though an explicit test following the second experiment showed evidence for AG learning in the human participants (Fig. S1). Thus, the human participants in this experiment were tested using a two‐alternative, forced‐choice experiment, in which they were presented with the testing sequences in a random order, and asked to respond whether the sequence ‘followed the same pattern’ as the exposure sequences or whether it ‘violated the pattern’. The human results are plotted as the proportion of trials on which the participants responded that the testing sequence violated the AG (see Materials and methods).

**Figure 4:**
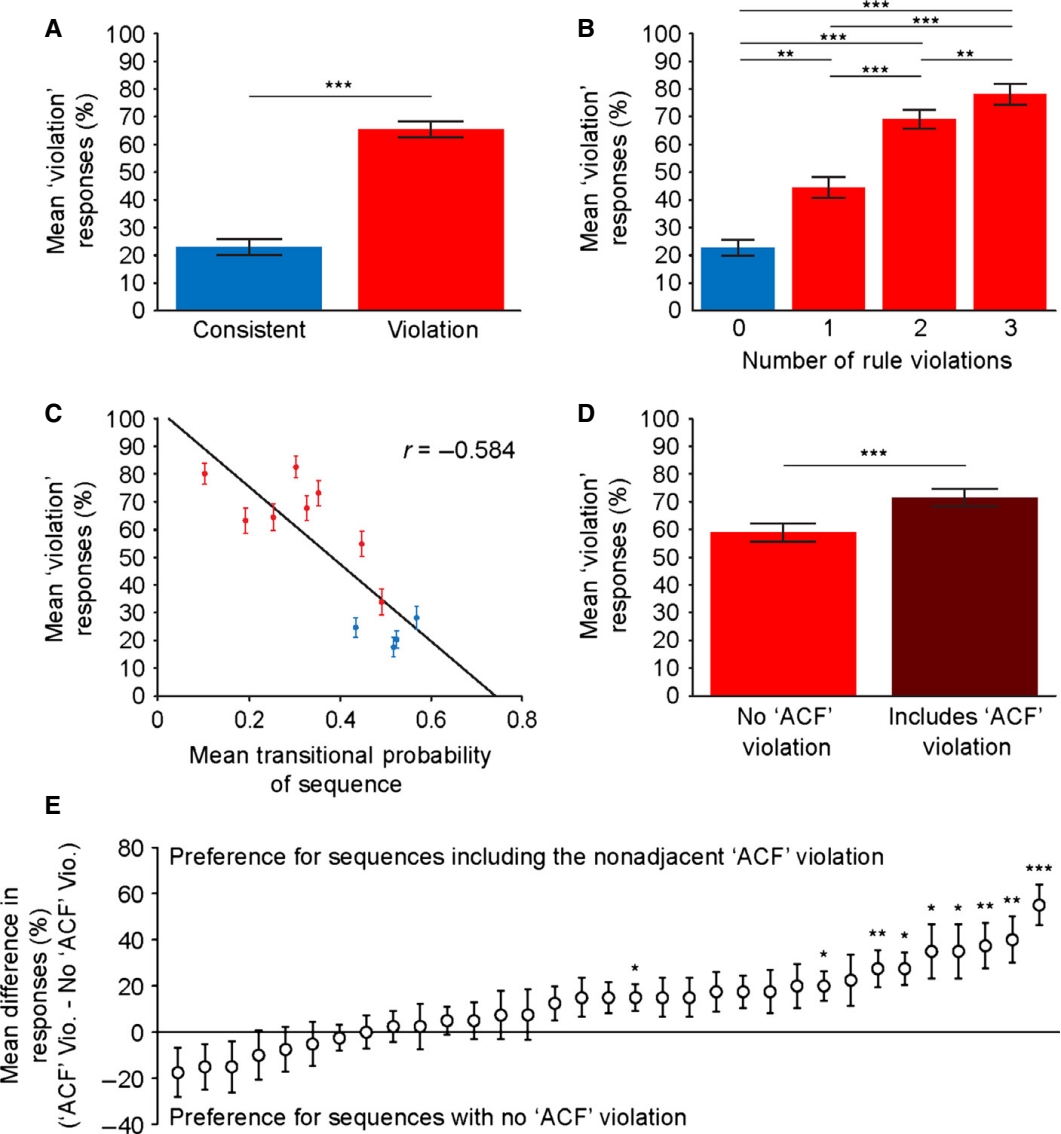
Human experiment results. (A) Mean (± SEM) proportion of trials on which participants gave the ‘does not follow the pattern’ (violation) response to the consistent and violation testing sequences. Values > 50% (chance level) represent accurate identification of the violation sequences (red), and values below 50% are accurate identification of the consistent sequences (blue). (B) Mean (± SEM) proportion of trials on which participants gave the ‘violation’ response separated based on the number of rule violations in the consistent (blue) or violation (red) sequences. (C) Mean (± SEM) proportion of trials on which participants gave the ‘violation’ response plotted against the mean transitional probability (TP) of each consistent (blue) and violation (red) sequence. (D) Mean (± SEM) proportion of trials on which participants gave the ‘violation’ response to violation sequences that only contained local violations, but not long‐distance, non‐adjacent ‘ACF’ violation (red), relative to those that also violated the long‐distance ‘ACF’ association in addition to local violations (dark red). (E) Mean (± SEM) difference in proportion of trials on which individual participants (ranked by performance) gave the ‘violation’ response to sequences containing the ‘ACF’ violation relative to those with no ‘ACF’ violation. Values higher than zero represent accurate identification of the sequences containing the ‘ACF’ violation. A paired‐samples sign test was performed for each participant, to identify those who responded to the non‐adjacent ‘ACF’ violation significantly above chance. *P <* *0.05, **P <* *0.01, ***P <* *0.001.

Like the macaques, the human participants produced significantly different responses to violation relative to consistent sequences (paired‐samples *t*‐test, *t*_32_ = 8.014, *P *<* *0.001; Fig. 4A). This shows that human participants can identify the sequences that violate the AG relative to those that do not. As in the macaques, we conducted an RM‐anova to investigate differences in responses to the familiar consistent, novel consistent and violation sequences (with the levels: familiar, novel, violation). As expected, we saw a strong main effect of ‘sequence condition’ *F*_2,64_ = 56.077, *P *<* *0.001. Bonferroni‐corrected *post hoc* tests revealed that, like the macaques, the human participants also responded differently to the violation sequences relative to both the familiar (*P *<* *0.001) and the novel (*P *<* *0.001) consistent sequences. There was a significant difference between the familiar and novel consistent sequences in the human participants (*P *=* *0.017), suggesting that the humans recognised the novelty of the sequences and that their behavioural performance benefited from this somewhat. Although this effect was not significant in the macaques, a qualitatively similar pattern of results was observed in the responses of both species (Fig. S2).

An RM‐anova including the factor: ‘number of rule violations’ (four levels) was performed to investigate how the human participants’ responses varied with the number of rule violations in the sequences. There was a strong main effect of number of rule violations (*F*_3,96_ = 54.932, *P *<* *0.001; Fig. 4B), showing that the human participants respond more strongly to sequences containing a greater number of rule violations. Furthermore, *post hoc* tests showed significant differences in responses between all of the different numbers of rule violations (*P *<* *0.01, Bonferroni corrected in all cases; Fig. 4B). This result shows that the human participants, like the macaques, are sensitive to the number of rule violations in a sequence. In addition to the number of rule violations in a sequence, the proportion of ‘violation’ responses given by the participants had a strong, negative correlation with the mean TP of the sequences (*r *= −0.584, *P *<* *0.001; Fig. 4C), showing that human participants are also sensitive to the statistical properties of the sequences.

Next, the responses to sequences that violated the non‐adjacent ‘ACF’ relationship relative to matched adjacent relationships were compared (Fig. 1B). An RM‐anova with the factors: ‘sequence type’ (‘ACF violation’ or ‘no ‘ACF’ violation’) and ‘sequence pair’ (four levels) was performed to investigate ‘ACF’ rule sensitivity in the human participants. There was a significant main effect of ‘sequence type’ (*F*_1,32_ = 18.103, *P *<* *0.001; Fig. 4D) and of ‘sequence pair’ (*F*_3,96_ = 27.477, *P *<* *0.001), as well as an interaction between ‘sequence type’ and ‘sequence pair’ (*F*_3,96_ = 3.429, *P *=* *0.02), suggesting that sensitivity to the ‘ACF’ violation was stronger in sequences with fewer adjacent rule breaks. These results imply that, in addition to recognising violations of adjacent relationships in the AG, the human participants also showed significant sensitivity to violations of the non‐adjacent ‘ACF’ relationship.

However, the human group results are insufficient to determine whether this effect represents a consistent sensitivity to the non‐adjacent violation in all of the participants or whether it is primarily driven by some individuals. Therefore, for each human participant we plotted the difference in responses between the sequences containing violations of both the adjacent and the non‐adjacent ‘ACF’ relationships, and those that contained only adjacent violations (Fig. 4E). In order to assess which participants showed statistically significant sensitivity to violations of the ‘ACF’ relationships, we compared the mean response to each sequence pair (with and without the ‘ACF’ violation, four pairs) for each testing run in each participant (each participant took part in five testing runs). Therefore, for this analysis 20 pairs of values per participant were entered into a paired‐sample sign test analysis. The results show that nine of the 33 participants (27%) have a significant sensitivity to sequences containing the ‘ACF’ violation (*P *<* *0.05; see Fig. 4E). These results suggest that while some participants are sensitive to the non‐adjacent violation, a large number of participants, like the monkeys, show comparable responses to the violation sequences with and without the non‐adjacent ‘ACF’ violation.

## Discussion

In this study we compared the sensitivity of macaques and humans to multiple features of a mixed‐complexity AG. We presented the participants with sequences containing transitions that occurred with varying probabilities, including violations of both adjacent and non‐adjacent relationships. This approach helped to identify similarities and differences in the patterns of behavioural responses within and across the species. We have previously used this AG to study the AG learning abilities of macaque and marmoset monkeys (Wilson *et al*., 2013). The present study compared the abilities of macaques and humans in greater detail, to assess whether these two species use similar or different AG processing strategies. We next consider the results obtained, their interpretation in light of differences in the procedures used to test the two species, and how behavioural insights such as these can inform us on the neurobiological processes that support AG learning in human and non‐human animals.

### Comparative human and macaque mixed‐complexity AG learning

The human and macaque results show that, after a period of exposure to exemplary consistent sequences generated by the AG, both species responded differently to violation relative to consistent testing sequences. These main effects suggest that both species are sensitive to some aspects of the AG sequences. In both species, this main effect persisted when responses to the violation sequences were compared with novel consistent sequences that were not presented in the exposure phase. This is consistent with a previously reported finding using a similar paradigm, where it is noted that the effects in Rhesus macaques cannot be easily attributed to rote memorisation or sequence familiarity (Wilson *et al*., 2013).

Beyond a general sensitivity to violations of the AG, the testing sequences in the current experiment were designed to allow us to investigate the sensitivity of human and non‐human primates to a wider range of the features present in this mixed‐complexity AG than has previously been possible (Saffran *et al*., 2008; Wilson *et al*., 2013). By varying the number of rule violations in the testing sequences, we were able to show that both the macaques and the human participants appeared to respond more strongly to sequences containing higher numbers of violations. In the humans a linear increase in performance was seen as the number of rule violations in a sequence increased from zero to three (Fig. 4B). The macaques also responded for longer durations to sequences with higher numbers of rule violations, although sequences containing two illegal transitions were sufficient to elicit the maximum responses observed in the macaques (Fig. 3C and D).

In addition to containing a range of explicit rule violations, we designed our testing sequences to contain transitions that occurred with a range of probabilities. We found strong negative correlations between the responses of both species and the average TPs of the sequences. This suggests that, along with the sensitivity to the number of rule violations, noted above, both monkeys and humans were sensitive to the frequency with which different transitions occurred in the exposure phase of the experiment.

Finally, our testing sequences were designed in pairs, in which both sequences contained matched adjacent violations, but an additional non‐adjacent ‘ACF’ violation was also present in one of the comparison sequences (Fig. 1B). Neither of the two macaques tested showed significant differences in response to these pairs of sequences, providing no clear evidence that they were sensitive to the non‐adjacent ‘ACF’ relationship. Instead, the results suggest that they primarily responded to what appear to be the more salient adjacent violations. By contrast, at the group level the human participants more accurately identified sequences containing the additional non‐adjacent violation, suggesting that they were sensitive to this non‐adjacent relationship. However, when the human participants were considered individually, it was apparent that many (73%) did not appear to notice the ‘ACF’ relationship. This suggests that, like the macaques, many of the humans may not notice the non‐adjacent violations over and above the adjacent violations in the sequences.

The results of these experiments highlight notable similarities in the responses of the macaques and human participants. Both species appear to notice sequences that violate the AG, particularly those containing higher numbers of rule violations and lower TPs. However, the responses to the sequences containing non‐adjacent violations suggest that unlike the monkeys, at least a subset of the human participants was sensitive to these non‐adjacent relationships.

### Relationship of current results to others in the literature

The current experiment allowed us to evaluate the learning of both adjacent and non‐adjacent AG relationships in parallel in the same mixed‐complexity AG. There is considerable evidence that non‐human animals are able to recognise violations of adjacent relationships in a range of AGs and experimental paradigms (Fitch & Hauser, 2004; Gentner *et al*., 2006; Murphy *et al*., 2008; Saffran *et al*., 2008; Hauser & Glynn, 2009; van Heijningen *et al*., 2009; Abe & Watanabe, 2011; Stobbe *et al*. 2012; Wilson *et al*., 2013). Many of these studies tested whether various species were able to recognise patterns based on two categories of stimuli (A and B). For example, in some studies the two categories were syllables produced by male or female speakers (Fitch & Hauser, 2004), tones of two different pitches (Murphy *et al*., 2008), or different categories of conspecific vocalisations (Gentner *et al*., 2006; Hauser & Glynn, 2009; van Heijningen *et al*., 2009). These studies have shown that non‐human primates, songbirds and rodents are sensitive to the relationships between adjacent stimuli, and that they can identify sequences that violate a previously learned pattern (e.g. AAB, ABA or ABAB). By contrast, a number of other studies, including the current one, have used AGs that do not require explicit categorisation, as in the original AG study by Reber (1967). Such AGs generate sequences consisting of several different elements, which can occur in a wider range of orders and with a range of probabilities (Saffran *et al*., 2008; Abe & Watanabe, 2011; Wilson *et al*., 2013). These AGs can generate sequences that tend to be less predictable (non‐deterministic), such that there is considerable variability in the legal transitions allowed in a sequence. Studies using these sorts of AGs have shown that non‐human animals are sensitive to violations of various sorts of adjacent relationships between sequence elements.

Therefore, there is converging evidence that different non‐human animals are sensitive to adjacent relationships in a range of AGs. However, non‐adjacent relationships present an increase in sequence‐processing complexity (Petkov & Wilson, 2012), and fewer studies have reported on the extent to which non‐human primates, in particular, are sensitive to long‐distance, non‐adjacent AG relationships. Newport *et al*. (2004) demonstrated that tamarin monkeys were able to learn the non‐adjacent relationship between the first and third syllable of a three‐syllable sequence. More recently, spider monkeys have been shown to be sensitive to violations of tone sequences of the form AB^*n*^A (i.e. two A elements separated by a varying number of B elements; Ravignani *et al*., 2013). That study reported that the monkeys were sensitive to the non‐adjacent relationship between the first and last element of the sequences. Tamarin monkeys have been shown to learn an adjacent relationship between alternating A and B stimulus categories (Fitch & Hauser, 2004). However, the same study reported that the tamarin monkeys were not able to recognise violations of more complex sequences that included non‐adjacent relationships between the A and B stimulus categories in sequences of the form A^*n*^B^*n*^ (e.g. AAABBB; Fitch & Hauser, 2004). These sets of studies suggest that non‐human primates may be able to learn certain types of non‐adjacent relationships, but that it might be more difficult to measure their sensitivity to these relationships than to adjacent relationships.

The present study is, to our knowledge, the first to comparatively evaluate human and non‐human primate sequence‐processing behaviour using a mixed‐complexity AG containing both adjacent and non‐adjacent relationships. In a recent human study, Romberg & Saffran (2013) tested adult human participants with a mixed‐complexity AG. The authors found that although participants did appear to be able to learn both adjacent and non‐adjacent relationships simultaneously, they only correctly identified violations on ~60% of trials (with chance levels at 50%). These observations suggest that mixed‐complexity AGs are challenging even for human participants to learn. Therefore, it remains possible that the presence of adjacent relationships in mixed‐complexity AGs may overshadow the non‐adjacent relationships also present. Indeed, studies in human adults and infants have demonstrated that non‐adjacent rule learning typically occurs only when adjacent relationships are unpredictable and uninformative (Gomez, 2002).

### Interpretations informed by differences in how the species were tested

We now consider how the choice of experimental design, stimuli and how the two species were tested inform the interpretation of the results and provide directions for future study. As in many studies in non‐human primates (Fitch & Hauser, 2004; Saffran *et al*., 2008), the macaques in this experiment were given substantially more exposure to the consistent sequences than the humans. One might predict that this extra exposure time could have led to better learning in the macaques, including the non‐adjacent relationships. However, the length of exposure in both species seemed to be sufficient for both to show substantial and largely comparable sensitivity to the adjacent relationships, but not necessarily the non‐adjacent relationship. It is possible that additional exposure may have helped the macaques and more of the humans to learn the non‐adjacent ‘ACF’ relationship. However, if the presence of the adjacent relationships overshadows the less salient non‐adjacent relationships in this mixed‐complexity AG, further exposure might make little difference.

It is also possible that the use of nonsense word stimuli may have offered some of the human participants an advantage in identifying the non‐adjacent relationship. However, the monkeys were able to distinguish the nonsense words sufficiently well to evaluate their relationships in a sequence, as shown by their differential responses to the consistent and violation AG sequences. The nonsense word stimuli in this experiment were chosen as they are spectro‐temporally complex sounds, which were sufficiently interesting to elicit looking responses in the macaques. Finally, these sounds fall well within the audible hearing ranges of both humans and macaques, which are reasonably comparable at these frequencies (Fig. S3).

In the current experiments, Rhesus macaques were tested using an eye‐tracking paradigm while human participants took part in a two‐alternative forced‐choice task. We conducted two eye‐tracking experiments in adult human participants. However, these experiments failed to provide any evidence of AG learning, despite evidence from a brief two‐alternative forced‐choice experiment at the end of the second eye‐tracking experiment that AG learning had occurred in the participants (Fig. S1). Thus, like many previous studies (Fitch & Hauser, 2004), we opted to test the humans and macaques using different methods, in what seems to be the most natural way for each of the species. It is possible that the use of a forced‐choice paradigm relative to a free‐looking, eye‐tracking experiment might have encouraged the human participants to attend to the stimuli more strongly. This could have given some of the human participants an advantage in noticing the non‐adjacent relationship in this AG. However, even with such an advantage, it is interesting that the majority of human participants did not show a significant sensitiv‐ity to the non‐adjacent relationship. The differences in testing approaches notwithstanding, the patterns of results in macaques and humans are strikingly comparable. This is particularly evident in their response to the adjacent AG relationships, suggesting that both species appear to use similar learning strategies for processing these aspects of the AG.

### Behavioural insights informing neurobiological data on AG learning

AG learning paradigms and other auditory tasks, which can assess the processing of sequences of different levels of complexity, are supported by neurobiological processes involving auditory cortex and hierarchically higher brain areas. For example, human neuroimaging studies have shown that, following exposure to exemplary AG sequences, violation sequences can engage perisylvian brain regions around the Sylvian or lateral sulcus (Friederici, 2011). Whether certain regions in this network are activated depends on the complexity of the sequences (Friederici *et al*., 2006; Bahlmann *et al*., 2008). For instance, relatively simple oddball tasks that present an infrequent ‘deviant’ sound in a repeated stream of ‘standard’ sounds (Bekinschtein *et al*., 2009) can elicit a mismatch negativity electroencephalogram (EEG) response in humans and other animals (Javitt *et al*., 1992; Naatanen & Alho, 1995; Bekinschtein *et al*., 2009; Honing *et al*., 2012). This enhanced negativity at ~150 ms is thought to involve neurons in the auditory cortex responding more strongly to unexpected stimuli (Ulanovsky *et al*., 2003; Fishman & Steinschneider, 2012). More complex oddball paradigms have repeatedly presented a short sequence of tones, in which a different pattern of tones is the oddball, deviant stimulus. The sequences that deviate from the standard pattern can engage a distributed dorsal fronto‐parietal network, thought to be involved in general deviance detection (Bekinschtein *et al*., 2009; Uhrig *et al*., 2014). Regions of the human ventral frontal cortex, which are also involved in natural language processes, can be activated by more complex sequence‐processing tasks or AG learning paradigms, especially those that create longer, more non‐deterministic sequences, and/or contain non‐adjacent relationships (Petersson *et al*., 2004; Friederici *et al*., 2006).

As sequences become longer and less predictable and contain violations that can occur at any point in the sequences, there becomes a greater need for clarity on the behavioural strategies that an individual could employ (Gentner *et al*., 2006; van Heijningen *et al*., 2009). On one hand, one can create a very specific illegal transition in a sequence that, apart from this violation, is identical to a matched legal ‘consistent’ AG sequence. We used this strategy in a recent macaque EEG experiment to identify event‐related potentials in response to the illegal violation (Attaheri *et al*., 2014). Multiple violations can elicit stronger behavioural responses (Figs 3C and D, and 4B) and scale with functional magnetic resonance imaging (fMRI) activity in the ventral frontal cortex in both macaques and humans (Wilson *et al*., 2011). However, in these cases it becomes important to study the behavioural responses across the species and in individual participants in more detail, in order to better relate patterns of fMRI activation to specific AG processing strategies. For instance, in a comparative fMRI study using the current AG, both humans and macaques appear to show comparable activation in regions of the ventral frontal cortex (BA 44/45; Wilson *et al*., 2011). Comparable patterns of activation would be consistent with the similarities shown here in the behavioural responses across the species for processing the adjacent relationships in the AG. However, any differences in patterns of fMRI activation between the species could represent either evolutionary divergences in neurobiological processes or might stem from different auditory sensitivities or sequence learning strategies. Therefore, although the comparative neurobiological study of AG processing is still a developing field, intriguing cross‐species similarities and differences in brain regions and processes associated with AG learning could potentially be better understood in light of comparative behavioural research.

## Conclusions

Our human and macaque results suggest that in the presence of multiple cues to ‘grammaticality’, both species are largely comparably sensitive to violations of the adjacent relationships. Both macaques and humans seem to respond more strongly to sequences containing higher numbers of rule violations and lower TPs. Although these results do not provide evidence that the macaques learned the non‐adjacent ‘ACF’ relationship, it is notable that most humans also did not notice this relationship. Given that it was not possible to use the same testing methods in the two species, it is striking how comparably both species responded to the violations of the adjacent relationships in the AG, which suggests that overall the two species use similar auditory sequence learning strategies.

## Author contributions

B.W. and C.I.P. conceived and designed the experiments; B.W. performed the experiments; B.W. analysed the data; B.W., K.S. and C.I.P. contributed reagents/materials/analytic tools; B.W., C.I.P. wrote the paper (with input from K.S.).

## Supporting information

Fig. S1. Eye‐tracking experiments testing AG learning in adult human participants.
Fig. S2. Responses to familiar and novel consistent testing sequences, relative to violation sequences, in the macaques and humans.
Fig. S3. Nonsense word power spectra in relation to human and macaque audiograms.
Table S1. Stimuli for human eye‐tracking experiments.
Data S1. Responses to the first presentation of each consistent testing sequence.
Data S2. Analyses of responses across testing runs.
Data S3. Analyses of human participants’ reaction times.
