# Supplementary material for: Mixed‐complexity artificial grammar learning in humans and macaque monkeys: evaluating learning strategies
Source: Eur J Neurosci. 2015 Mar 1;41(5):568–78. doi: 10.1111/ejn.12834 (PMC4493314; doi:10.1111/ejn.12834)
Supplement: Supplementary file 1 — Fig. S1. Eye‐tracking experiments testing AG learning in adult human participants. Fig. S2. Responses to familiar and novel consistent testing sequences, relative to violation sequences, in the macaques and humans. Fig. S3. Nonsense word power spectra in relation to human and macaque audiograms. Table S1. Stimuli for human eye‐tracking experiments. Data S1. Responses to the first presentation of each consistent testing sequence. Data S2. Analyses of responses across testing runs. Data S3. Analyses of human participants’ reaction times. [file EJN-41-568-s001.pdf]

Supplementary Materials for Wilson *et. al.*, '*Mixed-complexity artificial grammar learning in humans and macaque monkeys: Evaluating learning strategies*'

Supplementary Figure 1: Eye-tracking experiments testing AG learning in adult human participants

In the main article we report data from an eye-tracking experiment in macaques and from a two-alternative forced-choice experiment in humans. While it would be ideal to test the humans and macaques with the same methods (for further discussion of this point, see Petkov & Wilson, 2012), different species might find it more natural to respond in different ways, making using the same tasks difficult. Here, we report two eye-tracking experiments conducted in human participants. In the first experiment 11 participants were not given any instructions regarding where to look during the stimulus presentation. The results show that adult humans did not tend to look towards the speaker presenting the auditory AG sequences. In the second experiment we asked 10 different participants to try to localise the source of the testing sequences by looking towards it. In this experiment, on average participants did look towards the speaker for longer, but again the looking responses did not relate to whether the sequences were consistent with the AG or whether they contained violations. The same participants were then tested with a two-alternative forced-choice experiment like the one described in the article and showed evidence of having learned the artificial grammar. Thus neither of these two eye-tracking experiments were able to measure any effects of AG learning based on adult humans' natural looking responses.

Stimuli

The human eye-tracking experiments used the same AG as the main experiments reported here. The stimulus sequences and nonsense words were the same as those used in our previous study (see Suppl. Table 1 and Wilson *et al.*, 2013).

Supplementary Table 1. Stimuli for human eye-tracking experiments. Table adapted from Wilson *et al.*, (2013).

AG Element	Nonsense Word	Exposure Sequences	Testing Sequences	Condition
A	'klor'	ACF	ACGFC	Correct
C	'biff'	ACFC	ADCFCG	Correct
D	'jux'	ACGF	ACFCG	Correct
F	'cav'	ACGFC	ADCGFC	Correct
G	'dupp'	ACGFCG	AFGCD	Violation
		ADCF	AFCDGC	Violation
		ADCFC	FADGC	Violation
		ADCFCG	DCAFGC	Violation
		ADCGF		

Human eye-tracking experiment 1: Free looking

In the first human experiment we tested 11 participants (1 male, 10 female; age range 18-36 years). The participants were initially exposed to the testing sequences binaurally for 5 minutes, without explicit instruction about what they were listening to. As in the macaque experiment, in the subsequent testing phase, the participants were presented with a fixation spot for 2 seconds, before the spot disappeared and a stimulus was presented from one of two audio speakers located at $\pm 30^{o}$ visual angle concealed behind a black curtain. The participants were asked to fixate on the fixation spot while it was present, but were given no further instructions about where to look during the stimulus presentation. Eye-tracking data were recorded with the participants in an eye-tracking head frame using the same infra-red eye-tracking system that we used with the macaques (see article Methods). Eye-tracking data were recorded from the beginning of the fixation period until 5 seconds after the stimulus was presented (for a total of 7 seconds of eye-tracking data). Each participant took part in 5 testing runs (each containing 4 presentations of each stimulus). Each testing phase was separated by a re-familiarisation period during which the participant listened to the exposure sequences for 3 minutes prior to the start of the next testing run.

First, we plotted the participants' mean eye position relative to the presenting audio speaker (Suppl. Fig. 1A). The participants fixated on the spot accurately and on average, the participants did tend to look towards the presenting audio speaker, however these looking responses to the sound from the hidden audio speaker were weak. The mean eye position during the stimulus presentation was less than 1° visual angle. This suggests that the participants typically kept looking towards the centre of the screen, where the fixation spot had previously been displayed, rather than looking towards the audio speakers. As in the macaque experiment, we analysed the duration of the responses based on a response threshold calculated from the variability in the fixation period. The participants fixated very tightly during the fixation period so this threshold was lower than for the macaques; therefore small eye movements were considered as responses. However, there were no differences between the responses to the consistent and violation sequences ($t_{10} = 0.336$, $p = 0.744$, Suppl. Fig. 1B). We also calculated the mean horizontal eye position throughout the stimulus period. Again, there was no significant difference in eye position between responses to consistent and violation sequences ($t_{10} = 0.698$, $p = 0.501$, Suppl. Fig. 1C).

This experiment failed to provide any evidence for differences in responses, in either direction, between consistent and violation AG sequences, possibly because the adult humans did not make many looking responses towards the audio sequences. To address whether effects were limited because the humans were insufficiently motivated to look toward the speakers, we conducted a second experiment using identical stimuli and methods, in which we encouraged the participants to look towards the presenting audio speaker to localise the sounds.

Human eye-tracking experiment 2: Sound localisation

In this second eye-tracking experiment we modified the instructions the participants received in order to encourage them to make more looking responses. As in the first experiment the participants were asked to fixate on the centrally located fixation spot. When the fixation spot disappeared they were asked to localise the sound from the hidden speaker by looking towards it.

Other than these new instructions, the experiment and analyses were performed identically to the previous experiment. 10 participants were tested in this experiment (4 male, 6 female; age range 18-27 years).

In this experiment, the participants made much stronger looking responses towards the presenting audio speaker than in the previous experiment (Suppl. Fig. 1D). However, the participants did not respond for longer to either the consistent or violation sequences (t_9 = 1.02, p = 0.332, Suppl. Fig. 1E). Furthermore, there was no significant difference in eye position during the presentation of consistent vs violation sequences (t_9 = 1.63, p = 0.14, Suppl. Fig. 1F).

In summary, both of the eye-tracking experiments failed to provide any evidence from adult human looking responses that artificial grammar learning has occurred. To determine whether this result reflects a failure of these participants to learn the AG we also tested the participants of the second experiment with a two-alternative forced-choice task, like the one used in the main experiments here. Following the eye-tracking experiment each of the participants took part in a single testing run where they were explicitly asked if the testing sequences followed the same pattern as the exposure sequences or not. As in the main experiment, we found that the participants gave significantly more “violation” responses to the violation sequences (violation vs consistent; t_9 = 2.42, p = 0.03). This suggests that the participants were able to identify the violation sequences; however, unlike in the macaques, we were unable to measure their AG learning abilities using eye-tracking.

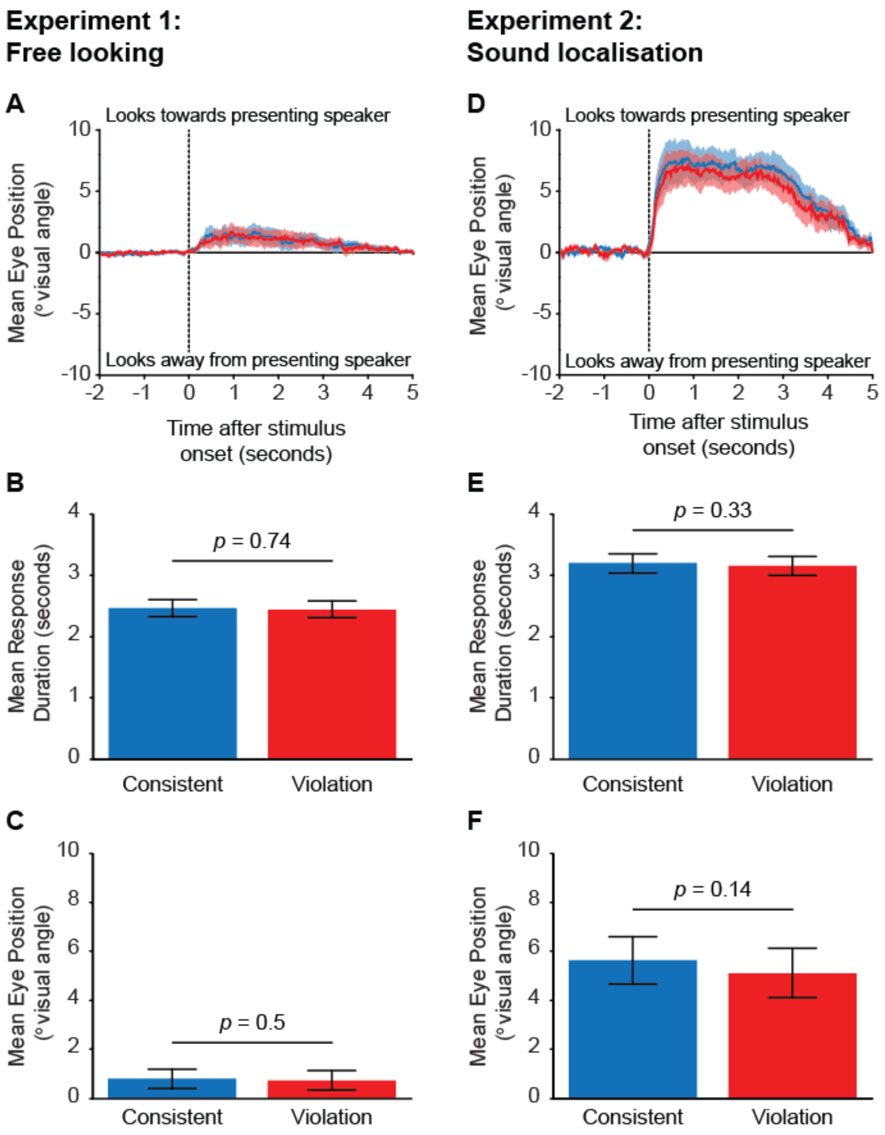

Supplementary Figure 1. Human eye-tracking experiments. (A-B) Mean (±SEM) eye position during the fixation period (from -2 to 0 seconds before stimulus presentation) and following the stimulus presentation (from 0-5 seconds). Responses to violation sequences are shown in red, those to consistent sequences are shown in blue. Positive eye positions represent looks towards the presenting speaker (whether the speaker was on the right or left of the monitor). (C-D) Mean durations (±SEM) of responses to the consistent and violation sequences, measuring the duration for which the eye position exceeded the threshold generated from variability in response in the baseline period (see Methods). (E-F) Mean (±SEM) eye position during the stimulus presentation period (0-5 seconds in A-B).

Supplementary Figure 2: Responses to familiar and novel consistent testing sequences, relative to violation sequences, in the macaques and humans

In the main analyses we noted that a significant difference was observed between the familiar and novel consistent sequences in the human participants, but not in the macaques (see Results). Evaluating the results more closely across the species suggests qualitatively similar patterns, although the human, but not the macaque results, are statistically significant (see article Results).

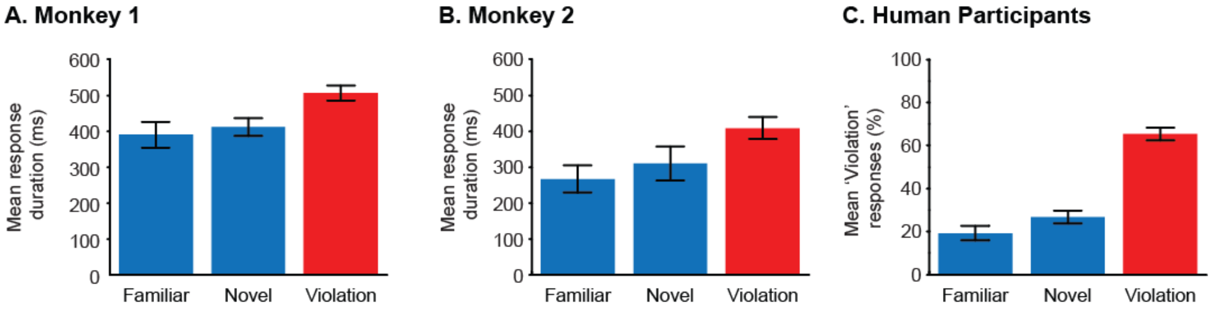

Supplementary Figure 2. Responses to familiar and novel consistent testing sequences, relative to violation sequences, in macaques and humans. (A-B) Mean (±SEM) response duration to the different testing conditions in the two macaques. (C) Mean (±SEM) percentage of 'Violation' responses in the human participants. In both of the monkeys and in the human participants, we observed stronger responses to the violation sequences than the consistent ones. In the human participants there was also a significant difference between the familiar and novel consistent sequences, which was not statistically significant in the macaques.

Supplementary Figure 3: Nonsense word power spectra in relation to human and macaque audiograms

The nonsense word stimuli in this experiment were designed to be well within the hearing range of both humans and macaques, which are very comparable based on published data. Suppl. Fig. 3 shows the average power-spectrum (±SEM) of the acoustic content in the 5 nonsense words. These stimuli were calibrated at an RMS sound level of ~75dB. On this plot we also show published data on human and macaque audiograms. The result shows that the stimuli were well within the audible range of both species, and that at least in this range, the stimuli were well within the audible range of the humans and macaques.

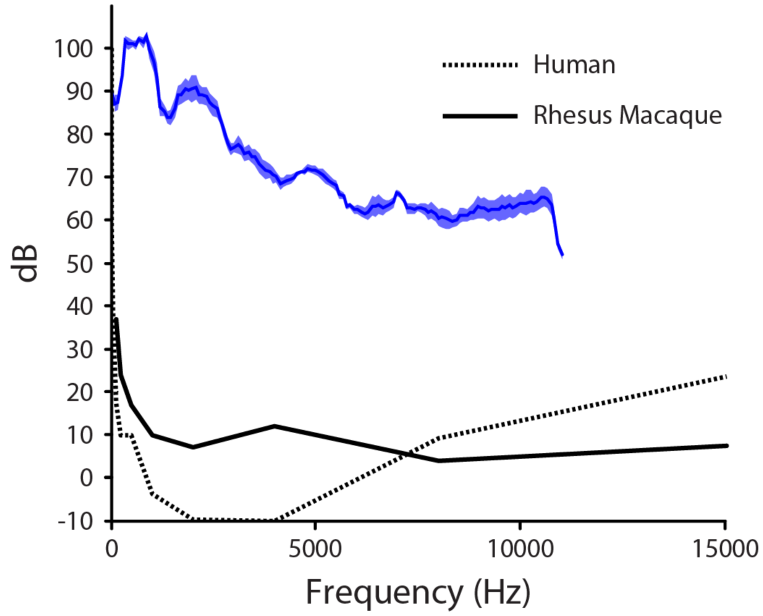

Supplementary Figure 3. Nonsense word power spectra in relation to human and macaque audiograms. Blue filled area signifies the range (±SEM) of the acoustic power spectrum of the nonsense word sounds (in dB re 20 µN/m^2). The black lines indicate the auditory threshold above which sounds at that frequency and intensity are audible for humans (dotted line) and macaques (solid line). The nonsense word stimuli fall well within the audible range of both the humans and macaques. Human audiogram data from: Jackson *et al.*, (1999). Macaque audiogram data summarised from: Pfingst *et al.*, (1975), Pfingst *et al.*, (1978), Lonsbury-Martin & Martin, (1981) and Bennett *et al.*, (1983)

Supplementary Text: Responses to the first presentation of each consistent testing sequence

In order to balance the number of presentations of the 8 violation testing sequences it was necessary to present each of the 4 consistent testing sequences twice each (Fig. 1). In order to assess whether the results reported in the article would persist when only the first presentation of each consistent sequence was considered, we reanalysed the data excluding the second presentation of each consistent sequence. Although omitting half of the consistent sequences reduces the power of the analyses, the results are comparable to those reported in the article.

An RM-ANOVA including the repeated measure of Condition (with two levels: consistent or violation sequence) and the between subjects factor of Monkey (two levels) produced a strong main effect of Condition ($F_{1,30}$ = 17.548, p < 0.001). There was no interaction between Condition and Monkey ($F_{1,30}$ = 0.303, p = 0.586). These observations show that the effects reported in the main experiment are also present when only the first presentation of each consistent sequence is considered. Next, we performed an RM-ANOVA including the three levels of familiar, novel and violation as a Condition factor. Again we saw a strong main effect of condition ($F_{2,30}$ = 9.01, p = 0.001) and no interaction between Condition and Monkey ($F_{2,30}$ = 0.441, p = 0.648). Bonferroni corrected post-hoc tests revealed a significant difference between familiar and violation sequences (p = 0.009) and a statistical trend towards a significant difference between the novel and violation sequences (p = 0.1). There was no significant difference between the familiar and novel sequences (p = 1.0). Although these results are underpowered relative to those in the article, they support the same conclusions. These results do not suggest that any differences between the consistent and violation sequences can be attributed to attenuated looking responses to the consistent sequences caused by the second repetition of these sequences.

Finally, we performed the same analyses on the human data. A paired samples t-test revealed that participants gave significantly more 'violation' responses to the violation sequences than to the first presentations of the consistent sequences (t_{32} = 8.03, p < 0.001). An RM-ANOVA including the factor Condition (with three levels: familiar, novel and violation sequences) showed a strong main effect of Condition ($F_{2,64}$ = 47.2, p < 0.001). Post-hoc tests revealed significant differences between the familiar and violation sequences (p < 0.001) and the novel and violation sequences (p < 0.001), but no difference between the familiar and novel consistent sequences (p = 0.367). These results recapitulate those in the main article.

Supplementary Text: Analyses of responses across testing runs

The monkeys participated in multiple testing runs in each testing session. To assess whether the monkeys might show diminished responses to the violation sequences after long sessions, we calculated the difference in response duration to the violation and consistent sequences for each testing run (violation – consistent) and correlated this with the number of testing runs the monkey had participated in that day. Neither monkey showed a significant correlation (M1: r = 0.327, p = 0.216; M2: r = 0.216, p = 0.572), demonstrating that the violation effect did not diminish on days with many testing runs. Furthermore, there was no correlation between the difference in response durations and the total number of testing runs the animal had participated in across all sessions (M1: r = -0.26, p = 0.922; M2: r = 0.121, p = 0.656). These analyses demonstrate that the monkeys' responses did not vary with the amount of testing they had received.

Additional analyses were performed to assess whether stronger responses to novel than familiar consistent sequences might have occurred in earlier testing runs but diminished with repeated testing. The difference between the animals' response durations to the novel and familiar sequences (novel – familiar) was calculated and correlated with the number of testing runs. There was no significant correlation between the responses to the novel relative to the familiar sequences and repeated testing in either animal (M1, r = -0.148, p = 0.583; M1, r = -0.217, p = 0.418). These results suggest that the monkeys' did not produce diminishing responses to the novel consistent sequences over multiple testing runs.

Supplementary Text: Analyses of human participants' reaction times.

Reaction times were recorded in the main human experiment. However, in order to ensure that the participants listened to the entire sequence before responding (rather than responding early and missing violations or important transitions later in the sequences), responses were only allowed after the entire sequence had been presented. Therefore, the reaction times do not clearly reflect how quickly participants recognised violations. Rather they reflect how quickly the human participants responded following the end of the sequence presentation.

The reaction time results were analysed in the same ways as the response data. A paired-sample t-test revealed no difference in RTs between the consistent and violation sequences (t_{32} = 0.423, p = 0.675). An RM-ANOVA revealed no significant relationship between RTs and the number of rule violations in the sequences ($F_{3,128}$ = 1.731, p = 0.164). There was also no correlation between RT and the mean TP of the sequences (r = 0.029, p = 0.500). Finally, there was no difference between violation sequences containing the 'ACF' violation relative to those that did not (t_{32} = 0.436, p = 0.666).

Supplementary References

Bennett, C.L., Davis, R.T. & Miller, J.M. (1983) Demonstration of presbycusis across repeated measures in a nonhuman primate species. *Behavioral Neuroscience*, **97**, 602-607.

Jackson, L.L., Heffner, R.S. & Heffner, H.E. (1999) Free-field audiogram of the Japanese macaque *(Macaca fuscata)*. *Journal of the Acoustical Society of America*, **106**, 3017-3023.

Lonsbury-Martin, B.L. & Martin, G.K. (1981) Effects of moderately intense sound on auditory sensitivity in rhesus monkeys: behavioral and neural observations. *J Neurophysiol*, **46**, 563-586.

Petkov, C.I. & Wilson, B. (2012) On the pursuit of the brain network for proto-syntactic learning in non-human primates: conceptual issues and neurobiological hypotheses. *Philos Trans R Soc Lond B Biol Sci*, **367**, 2077-2088.

Pfingst, B.E., Hienz, R. & Miller, J. (1975) Reaction-time procedure for measurement of hearing; Threshold functions. *Journal of the Acoustical Society of America*, **57**, 431-436.

Pfingst, B.E., Laycock, J., Flammino, F. & Lonsbury-Martin, B.L. (1978) Pure tone thresholds for the rhesus monkey. *Hearing Research*, **1**.

Wilson, B., Slater, H., Kikuchi, Y., Milne, A.E., Marslen-Wilson, W.D., Smith, K. & Petkov, C.I. (2013) Auditory artificial grammar learning in macaque and marmoset monkeys. *J Neurosci*, **33**, 18825-18835.
